# Efficiency of nilotinib to target chronic phase-chronic myeloid leukaemia primary mature CD34^−^ and immature CD34^+^ cells

**DOI:** 10.1038/s41598-021-85734-0

**Published:** 2021-03-17

**Authors:** Marc G. Berger, Benjamin Lebecque, Thomas Tassin, Louis-Thomas Dannus, Juliette Berger, Mélanie Soucal, Agnès Guerci, Pascale Cony-Makhoul, Hyacinthe Johnson, Gabriel Etienne, Denis Guyotat, Marie-Claude Gagnieu, Bruno Pereira, Sandrine Saugues, Olivier Tournilhac, Eric Hermet, Céline Bourgne

**Affiliations:** 1 Hématologie Biologique, CHU Clermont-Ferrand, Hôpital Estaing, 1 place Lucie et Raymond Aubrac, 63003 Clermont-Ferrand Cedex 1, France; 2 Equipe d’Accueil 7453 CHELTER, Université Clermont Auvergne, CHU Clermont-Ferrand, Hôpital Estaing, 1 place Lucie et Raymond Aubrac, 63003 Clermont-Ferrand Cedex 1, France; 3 CRB-Auvergne, CHU Clermont-Ferrand, Hôpital Estaing, 1 place Lucie et Raymond Aubrac, 63003 Clermont-Ferrand Cedex 1, France; 4 Hématologie Clinique, CHU Nancy, Hôpitaux de Brabois, Rue du Morvan, 54500 Vandoeuvre-lès-Nancy, France; 5 Hématologie Clinique, CH Annecy-Genevois, 1 Avenue de l’Hôpital, 74370 Metz-Tessy, France; 6 Institut d’Hématologie de Basse Normandie, CHU de Caen, Avenue de la Côte de Nacre, CS30001, 14033 Caen Cedex 9, France; 7 Hématologie Clinique, Institut Bergonié, 229 Cours de l’Argonne, 33076 Bordeaux Cedex, France; 8 Département d’Hématologie, Institut de Cancérologie Lucien Neuwirth, 108 Avenue Albert Raimond, 42270 Saint-Priest-en-Jarez, France; 9 Service de Biochimie et Biologie Moléculaire, UM Pharmacologie-Toxicologie, Groupement Hospitalier Sud, 165, chemin du grand Revoyet, 69495 Pierre-Bénite, France; 10 CHU Clermont-Ferrand, Délégation à la Recherche Clinique et à l’Innovation, 63003 Clermont-Ferrand, France; 11 Hématologie Clinique, CHU Clermont-Ferrand, Hôpital Estaing, 1 place Lucie et Raymond Aubrac, 63003 Clermont-Ferrand, France

**Keywords:** Cancer, Oncology

## Abstract

Accumulation in target cells is an essential pharmacokinetic step of targeted therapies. Tyrosine Kinase Inhibitors (TKI) against the BCR-ABL fusion protein in Chronic Phase-Chronic Myeloid Leukaemia (CP-CML) cells constitute a unique model in terms of efficacy, specificity, and in vivo demonstration of response heterogeneity by target cells. The overall therapeutic response to nilotinib is heterogeneous with no satisfactory explanation. To better understand the patients’ response heterogeneity, we quantified nilotinib uptake by primary CP-CML cells in standardized conditions using flow cytometry, which allowed also distinguishing mature (polymorphonuclear cells) from immature (CD34^+^) cells. Nilotinib was undetectable in 13.3% of PMN and 40% of CD34^+^ cells. Moreover, in CD34^+^ cells, intracellular nilotinib did not completely abolish BCR-ABL activity (monitored by CrkL phosphorylation inhibition), although nilotinib accumulated well in most CD34^+^ cell samples. Intracellular nilotinib concentration was inversely correlated with disease burden parameters, Sokal score, and early haematologic response at day 6 ± 1 only in PMN, suggesting an intrinsic ability to limit nilotinib entry in the forms with higher tumor cell burdenat diagnosis. These findings suggest that nilotinib accumulation in CP-CML cells is influenced by individual characteristics and intra-clonal heterogeneity, and might be used for pharmacokinetic studies and to assess the therapeutic response.

## Introduction

In the era of personalized medicine and targeted therapy, three steps are highly correlated with successful transfer from phase II to phase III trials: (i) drug exposure at the target site, (ii) binding to the pharmacological target, and (iii) functional modulation of the target and pharmacological response^1^. Each of these steps is delicate to evaluate, particularly the drug localization in the target cell subpopulation within a tissue. Indeed, due to the in vivo cell system complexity, discrepancies are often observed between the results obtained in vitro (biochemical tests) and in vivo (animal models or patients)^2^. Thus, to better understand the efficacy and/or toxicity of targeted therapies, it is crucial to develop methodologies that do not modify the drug molecule for monitoring its cellular accumulation in conditions as close as possible to the in vivo conditions^2^.

The first targeted therapy was developed for patients with Chronic Myeloid Leukaemia (CML), which is still considered a model for targeted therapy^3–5^. Specifically, the tyrosine kinase inhibitor (TKI) imatinib is an inhibitor of the BCR-ABL fusion protein that results from the translocation [t(9; 22)(q34; q11) (Ph^+^)]^6^. Imatinib has considerably increased the overall survival of patients with CML (8-year survival rate of approximately 85%) to similar levels as those of a group of age- and sex-matched controls^7,8^. This was followed by the development of second- and third-generation TKIs. However, despite their effectiveness, the close follow-up of patients showed that the therapeutic response is quite variable. Three main therapeutic responses can be described: (i) in most patients with CML (60–70%), disease is controlled, but long-term residual disease can be detected; (ii) in 10 to 30% of patients, response is optimal (> MR4.5) and TKIs can be stopped. However, only approximately 40 to 60% of these patients remain in remission after TKI withdrawal^9^; (iii) in a limited number of patients, CML is immediately resistant to TKIs. Moreover, despite the presence of the molecular target in all cancer cells and in all patients, individual variations in TKI susceptibility have been observed that are partly explained by patient-specific pharmacokinetic differences or target modifications (e.g. BCR-ABL mutations). In addition, a subpopulation of leukemic stem cells (LSC) is resistant to treatment in vivo, even after several years of TKI-based therapy, and is responsible for the relapses observed following TKI withdrawal after optimal response^10,11^.

Nilotinib is a second-generation TKI approved for the first-line treatment of patients with newly diagnosed chronic phase-CML. It is 30-fold more potent than imatinib^12^ and induces a faster and deeper therapeutic response^13–17^, although still variable among patients^18^. Unlike imatinib, nilotinib residual plasma concentration, which is considered to accurately represent the TKI pharmacokinetics, shows a considerable intra- and inter-patient variability and cannot be used in clinical practice^19–21^. Therefore, to identify the parameters involved in nilotinib ability to reach its target (BCR-ABL) inside the cells, several groups have evaluated the membrane pumps^22,23^, but with contradictory results that are difficult to translate in clinical practice^24^. Moreover, techniques for assessing the intracellular amount of nilotinib have been developed in order to take into account the different factors that influence its intracellular accumulation. However, these approaches do not take into account CML intra-clonal heterogeneity and cannot assess nilotinib targeting efficacy.

In this study, we adapted our flow cytometry procedure initially developed to quantify intracellular imatinib without modifying (e.g. tagging) the molecule^25^ in mature cells and CD34^+^ progenitors/LSC within the same CML sample, and then assessed BCR-ABL protein inhibition in function of nilotinib intracellular concentration.

## Results

### Measurement of nilotinib uptake by flow cytometry-based natural fluorescence detection

Like imatinib, nilotinib is a naturally fluorescent molecule under UV light^26^, thus making possible its detection by quantifying the UV fluorescence emission. Therefore, we exploited the flow cytometry method we developed to evaluate imatinib intracellular concentration^25^ to monitor nilotinib intracellular accumulation. As each cell population is characterized by its own natural fluorescence (i.e. auto-fluorescence due to endogenous fluorophores that become fluorescent when excited by UV light exposure), we compared cell samples incubated or not with nilotinib and quantified nilotinib intracellular concentration as the difference between the UV fluorescence of control and of treated cells (i.e. Additional Fluorescence Units; AFU) (Supplementary Fig. S1). In our hands, data for at least 500 target cells need to be acquired to calculate the mean fluorescence reliably. This number was reached for all samples used in this study.

**Figure 1:**
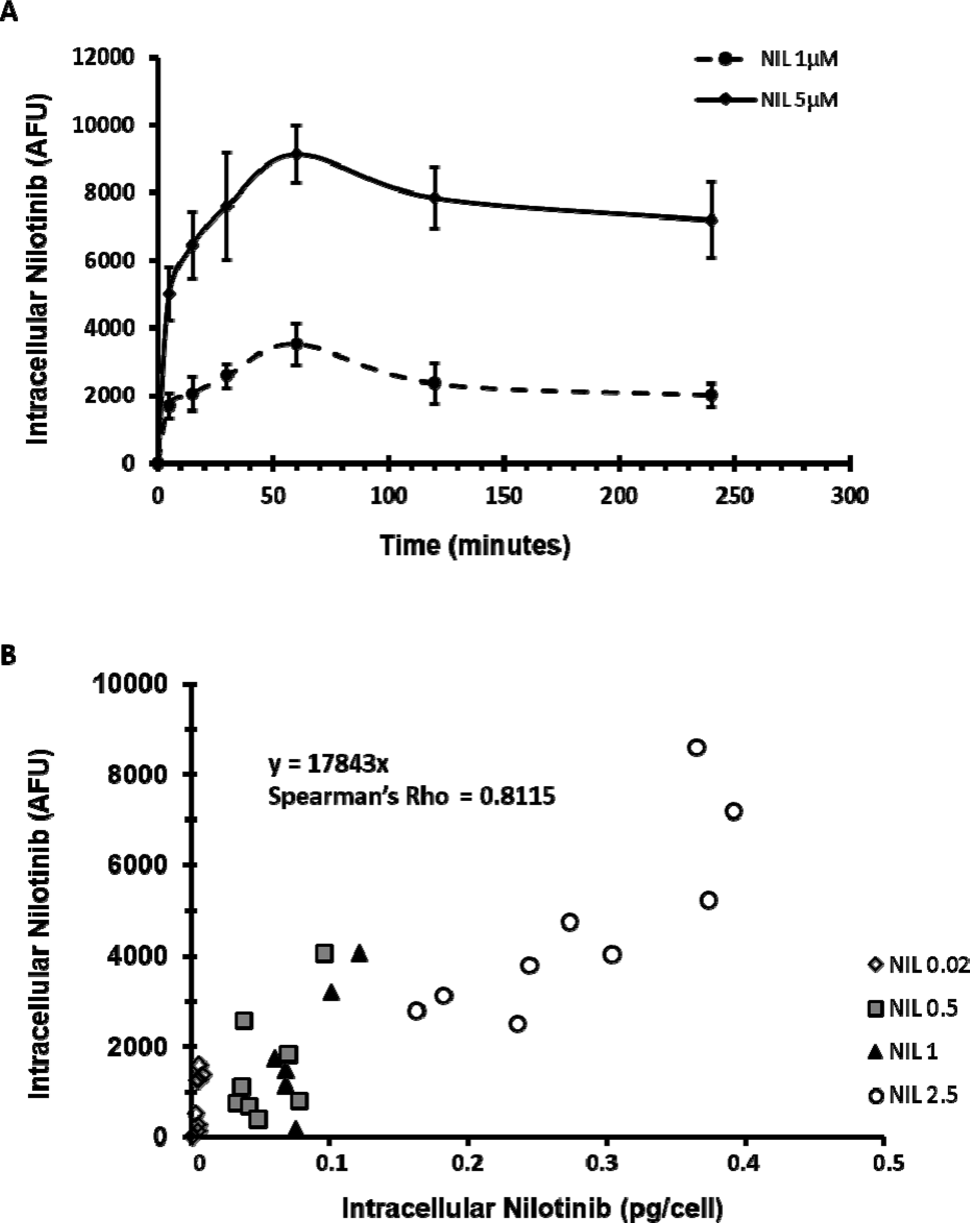
Evaluation of nilotinib uptake in K562 cells by flow cytometry. (**A**) Nilotinib uptake kinetics was evaluated in K562 cells incubated with 1 or 5 µM nilotinib at 5, 15, 30, 60, 120 and 240 min after TKI addition. The analysis showed a rapid nilotinib uptake followed by a plateau. Nilotinib intracellular concentration was defined as the additional fluorescence units relative to the fluorescence of control (untreated) cells. Data are the mean ± standard deviation of 4 experiments. (**B**) Correlation between the flow cytometry data (fluorescence) and nilotinib quantification by HPLC (pg/cell) after lysis of a known number of cells following incubation with nilotinib at the indicated concentrations. This confirmed that in our experimental conditions, the additional fluorescence (expressed as additional fluorescence units) is directly related to the amount of intracellular nilotinib (n = 39). At the lowest nilotinib concentration, nilotinib intracellular amount could be measured only by flow cytometry. NIL, nilotinib; AFU, additional fluorescence units.

We validated our approach by quantifying the UV fluorescence in K562 cells (derived from a patient with CML) at different time points (5, 15, 30, 60, 120 and 240 min) after incubation with 1 and 5 µM nilotinib (Fig. 1A). Nilotinib intracellular concentration (mean AFU) increased rapidly and in a dose-dependent manner after only 5 min of incubation: 1.7 × 10^3^ ± 0.38 and 5.0 × 10^3^ ± 0.79 for 1 and 5 µM nilotinib, respectively. After 2 h of incubation with nilotinib, AFU values remained stable. On the basis of these kinetic data, we used 2 h of incubation for all the experiments described here.

Then, we compared nilotinib uptake in three CML cell lines (K562, LAMA84 and KCL22 (Supplementary Fig. S2). On the basis of the linear relationship (r^2^ = 0.97) between nilotinib extracellular concentration and AFU values, we chose the K562 cell line for all subsequent experiments.

### Correlation between fluorescence values and nilotinib intracellular concentration assessed using a physical–chemical assay

To test the correlation between UV fluorescence emission (AFU) and intracellular nilotinib concentration (pg/cell), we analysed the correlation between AFU values measured by flow cytometry and amount of nilotinib released after lysis of a known number of cells from the same K562 cell suspension incubated with different nilotinib concentrations for 2 h (see Material and Methods). We found a significant correlation between these parameters (Spearman’s rho = 0.8115; *P* < 0.001; Fig. 1B). Specifically, 1 pg of nilotinib per cell was equivalent to 17.8 × 10^3^ AFU detected by flow cytometry. We then used this equivalence to express all data as pg of nilotinib per cell. However, in cells incubated with the lowest concentration of nilotinib (0.02 µM), we could detect nilotinib inside the cells only by flow cytometry, suggesting that this is a more sensitive technique.

### Nilotinib uptake by primary CML cells

**Figure 2:**
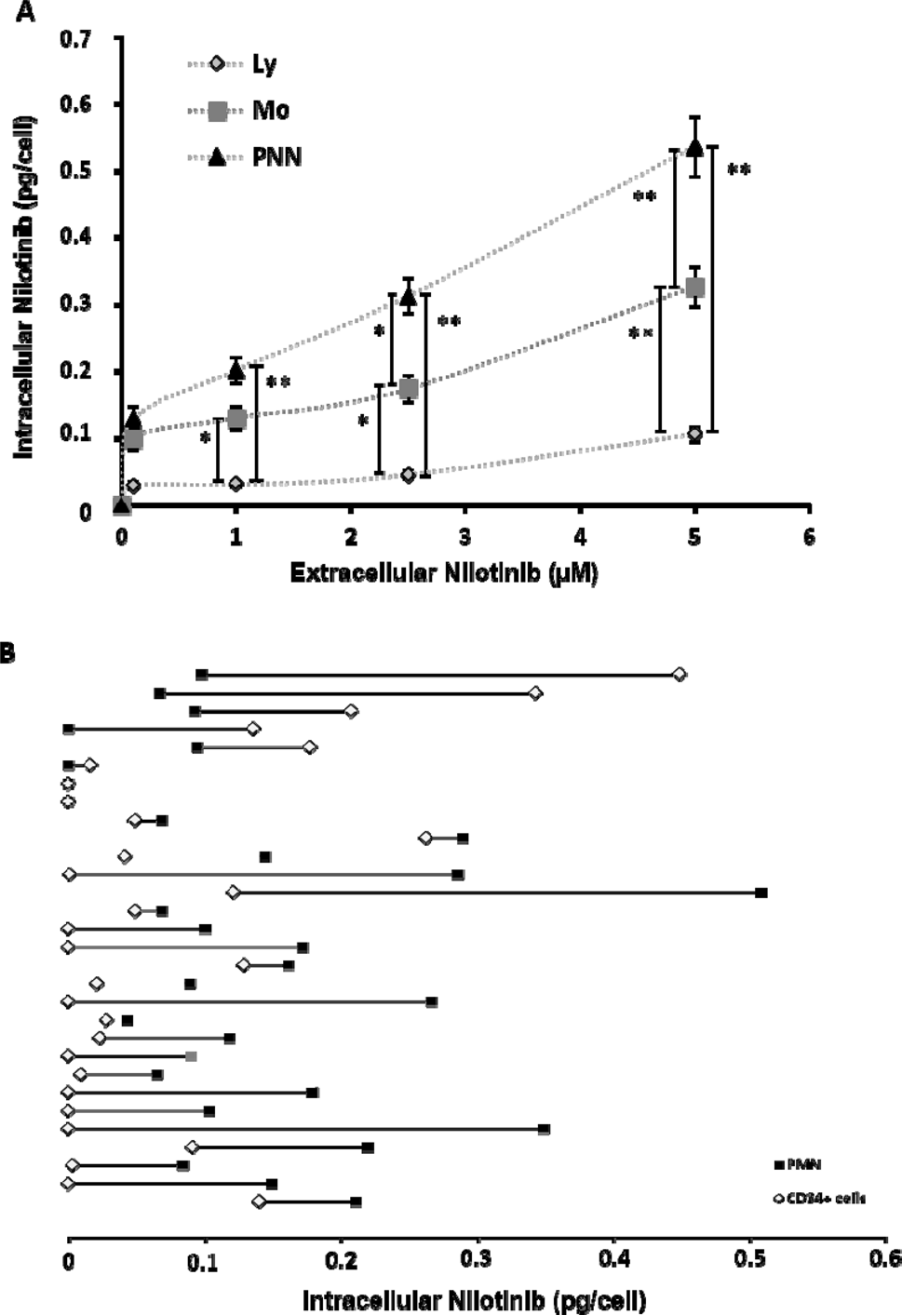
Nilotinib uptake in primary cells from patients with CML at diagnosis. (**A**) Nilotinib uptake by primary cells was evaluated by flow cytometry after 2 h of incubation with 0.1, 1, 2.5 or 5 µM of this TKI. Lymphocytes (Ly), monocytes (Mo), and polymorphonuclear cells (PMN) were identified on the basis of their FSC/SSC parameters. Nilotinib intracellular concentration was higher in PMN than in Ly and Mo (n = 60). Data are expressed as the mean ± standard deviation; the vertical bars indicate statistical comparisons, *P < 0.05, **P < 0.001 (**B**) Nilotinib intracellular amount quantification after identification by flow cytometry of immature CD34^+^ cells and mature PMN cells within the same sample. Nilotinib concentration was significantly lower in CD34^+^ than PMN cells (n = 30; *P* = 0.019), and was undetectable in CD34^+^ and PMN cells from 12 (40%) and 4 (13.3%) samples, respectively.

We then used our approach to analyse primary cells from patients with chronic phase-CML (Supplementary Table S1) at diagnosis before any TKI treatment (n = 92). All analyses were done only with cells isolated from peripheral blood. Nilotinib intracellular accumulation (AFU) was dose-dependent up to 5 μM (the highest tested concentration). Moreover, starting from 1 µM, nilotinib uptake was different in lymphocytes (Ly), monocytes (Mo) and polymorphonuclear cells (PMN) from the same sample (n = 60 patients; Fig. 2A and supplementary Table S2), as we previously showed for imatinib^25^. For instance, upon incubation with 2.5 µM of nilotinib, nilotinib intracellular concentration was significantly higher in PMN than in Mo (0.31 ± 0.003 *vs* 0.17 ± 0.02 pg/cell; *P* = 0.027) and Ly (0.03 ± 0.003 *vs* 0.05 ± 0.01 pg/cell; *P* < 0.0001). These results validated our strategy to assess in vitro nilotinib uptake by CML primary cells.

Moreover, flow cytometry allowed us to identify rare cell subsets without immunoselection, on the basis of the expression of specific cell surface markers. As in CML, LSC are in the CD34^+^ cell compartment, we could compare the in vitro uptake of nilotinib by mature CD34^-^ (PMN) and immature (CD34^+^) cells from 30 patients with CML (Fig. 2B). Nilotinib uptake by CML CD34^+^ cells was heterogeneous among patients, and was not correlated with the uptake by PMN. Overall, after 2 h of incubation with 1 µM nilotinib, its concentration in immature CD34^+^ cells was significantly lower than in mature PMN cells (0.08 vs 0.14 pg/cell respectively, *P* = 0.019). This difference was explained mainly by the undetectable level of nilotinib in CD34^+^ cells from 12 (40%) patients. Conversely, we could not detect nilotinib in PMN from four (13.3%) patients (this group included also two patients with undetectable nilotinib in CD34^+^ cells). In the 18 patients with detectable nilotinib in CD34^+^ cells, we did not observe any relationship between nilotinib uptake in CD34^+^ cells and in PMNs. Nilotinib concentration was higher in PMN than in CD34^+^ cells in 12 patients, and in CD34^+^ cells in 6 patients.

### Relationship between nilotinib uptake and in vitro BCR-ABL inhibition

**Figure 3:**
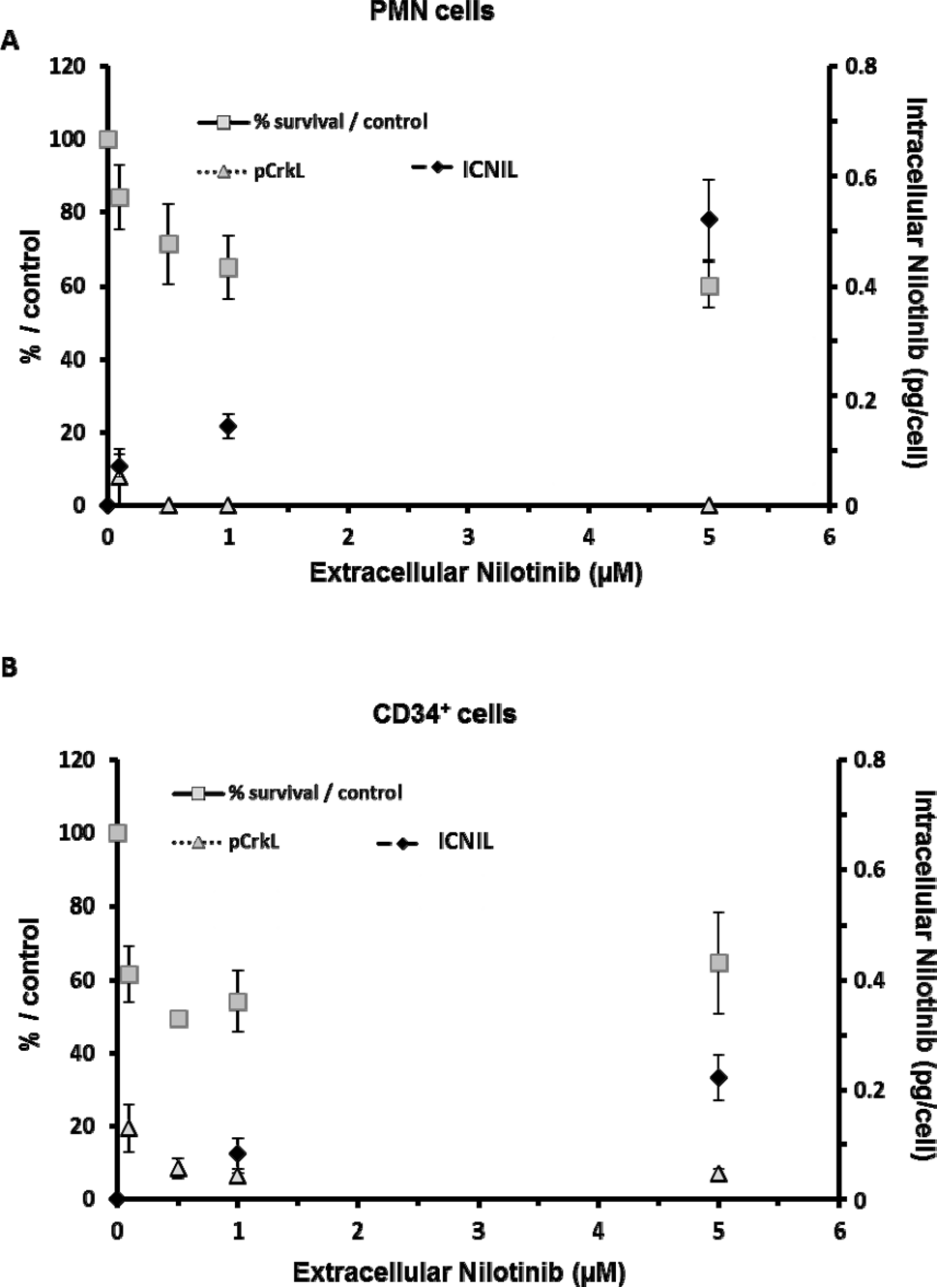
Relationship between intracellular nilotinib concentration and TKI efficiency in vitro. (**A**) The relationship between intracellular nilotinib (ICNIL; after 2 h of incubation) and nilotinib efficiency (i.e. inhibition of CrkL phosphorylation, pCrkL, and cell survival at 30 h of incubation) was evaluated in mature CD15^+^CD34^-^ (PNM) and (**B**) immature CD15^-^CD34^+^ cells (n = 3) after incubation with the indicated concentrations of nilotinib (extracellular nilotinib). Results are the mean ± standard deviation.

We then studied the relationship between nilotinib intracellular concentration and its targeting efficiency in primary CML cells (n = 3) by assessing the inhibition of CrkL phosphorylation (pCrkL), as a molecular target of BCR-ABL TK activity, and cell survival after 30 h of incubation with increasing nilotinib concentrations (Fig. 3A,B). CrkL phosphorylation in PMN and CD34^+^ cells was strongly decreased already after incubation with the lowest concentration of nilotinib. CrkL phosphorylation inhibition was complete in PMN from 0.5 µM of nilotinib, whereas a residual CrkL phosphorylation (about 10%) persisted in the immature CD34^+^ compartment, even in the presence of high intracellular amount of nilotinib (0.5 pg/cell). After 30 h of incubation with 1 µM of nilotinib (the clinical therapeutic plasma concentration), cell survival was comparable in PMN and CD34^+^ cells (65 ± 8% and 54 ± 8% of living cells relative to control, respectively).

### Relationship between nilotinib intracellular uptake and patients’ characteristics

We then evaluated the relationship between nilotinib intracellular uptake before treatment, Sokal prognostic score at diagnosis (low, intermediate and high risk), and features of disease burden (leucocytosis, number and percentage of circulating CD34^+^ cells) measured at diagnosis and at day 6 ± 1 days after treatment initiation.

**Figure 4:**
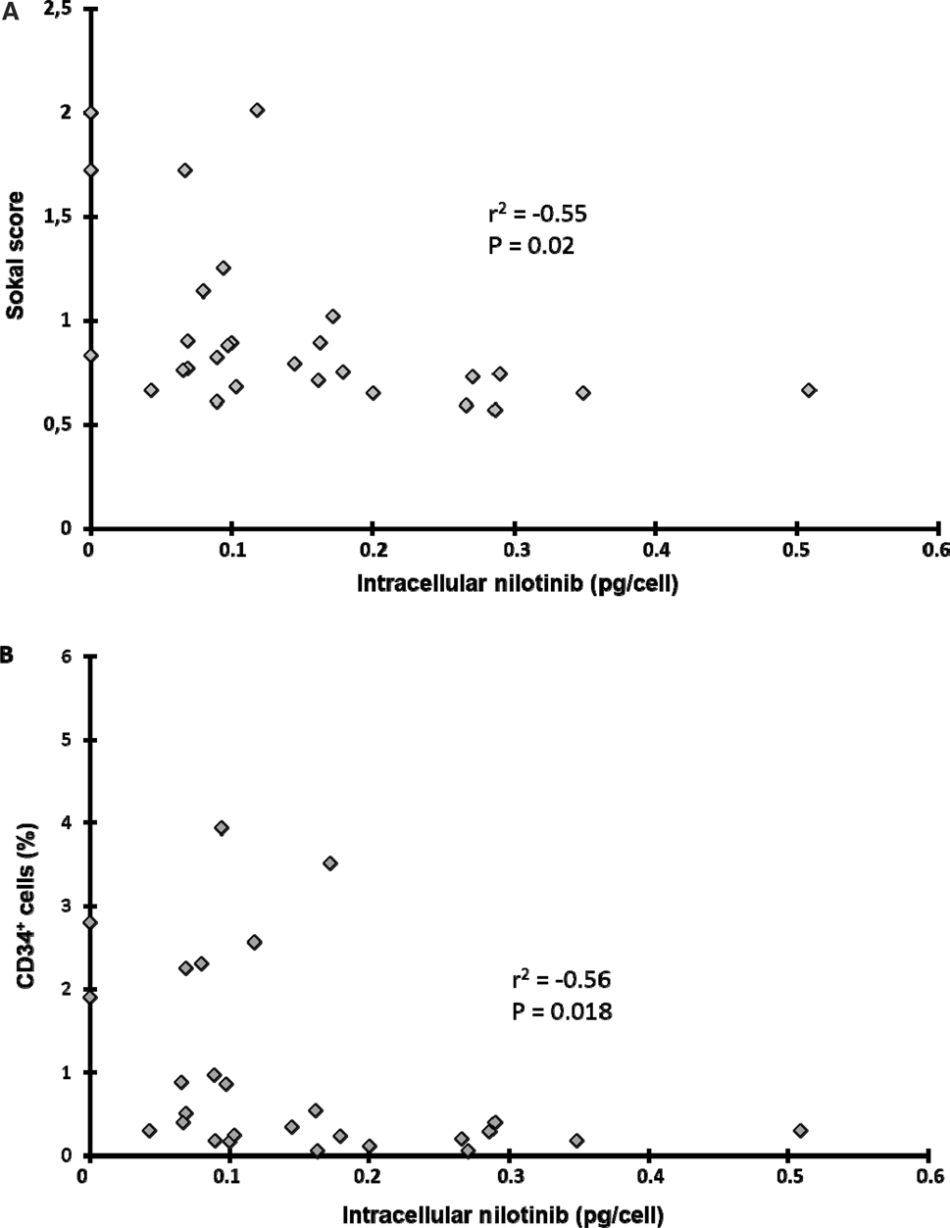
Relationship between intracellular nilotinib concentration in PMN cells and tumour burden in patients who received first-line treatment with nilotinib. Relationship between nilotinib concentration in mature PMN cells and (**A**) Sokal prognostic score at diagnosis and (**B**) percentage of CD34^+^ cells in peripheral blood (n = 25 patients).

We first evaluated nilotinib intracellular concentration in PMN from 28 of the 33 patients who received nilotinib as first-line treatment (Supplementary Table S3). After incubation with 1 µM nilotinib, the median intracellular concentration was 0.10 pg/cell (0–0.51). Nilotinib intracellular concentration was significantly and negatively correlated with Sokal prognostic score (*P* = 0.02) (Fig. 4A), percentage of CD34^+^ cells in peripheral blood (Fig. 4B; *P* = 0.018), and number of circulating CD34^+^ cells/µl (*P* = 0.03). Leucocytosis and percentage of CD34^+^ cells were lower in patients with higher nilotinib uptake by PMN (*P* = 0.04 and 0.05, respectively). We did not find any correlation between these parameters and nilotinib intracellular concentration in CD34^+^ cells.

**Figure 5:**
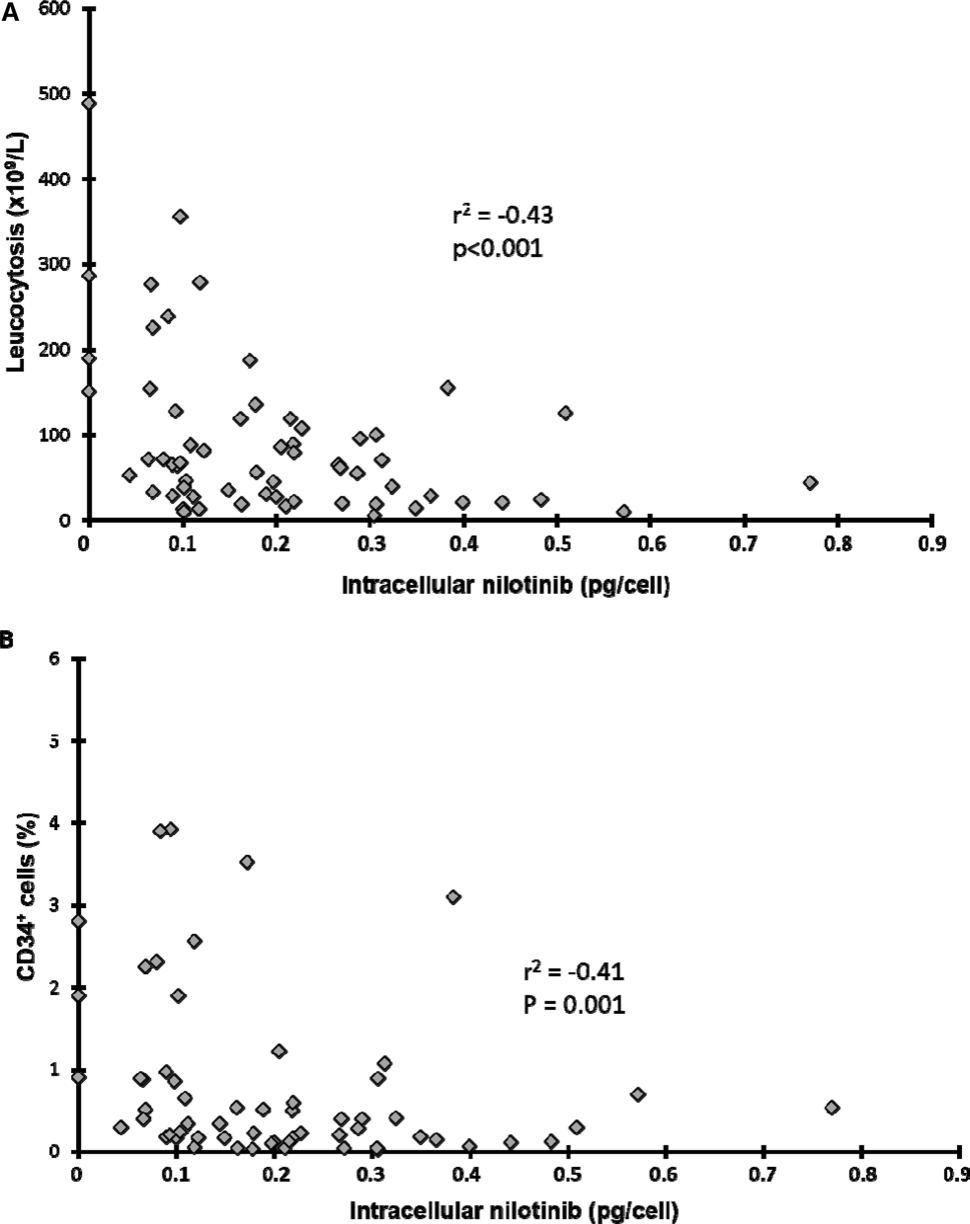
Relationship between intracellular nilotinib in PMN cells and tumour burden in the whole population. Relationship between nilotinib concentration in mature PMN cells and (**A**) leucocytosis and (**B**) percentage of CD34^+^ cells in peripheral blood (n = 61 patients).

In the total population (n = 92) (Supplementary Table S1), the median nilotinib concentration in PMN was 0.17 pg/cell (0–0.77) after incubation with 1 µM. We did not find any correlation between nilotinib intracellular concentration in PMN and Sokal prognostic score, whereas nilotinib intracellular concentration was inversely correlated with leucocytosis (Fig. 5A; *P* < 0.001), number of circulating CD34^+^ cells (*P* < 0.001), and percentage of CD34^+^ cells (Fig. 5B; *P* = 0.001).

Moreover, in the patients with undetectable nilotinib in CD34^+^ cells (n = 12/30 patients; Fig. 2B), the Sokal prognostic score at diagnosis was low in seven, intermediate in three, and high in two. Interestingly, in 8/12 patients (67%), BCR-ABL transcript level was higher than 1% (i.e. indicative of residual disease) after one month of TKI treatment. Conversely, among the patients with detectable nilotinib in CD34^+^ cells, BCR-ABL transcript level was higher than 1% in 7/18 patients (39%) after one month of TKI treatment.

Due to the absence of correlation between nilotinib uptake in CD34^+^ cells and in PMNs, we tried to summarize nilotinib uptake capacity by these two cell subsets in the same patient by calculating the ratio of the difference of the mean nilotinib concentration in CD34^+^ cells and in PMNs to the sum of the two concentrations (considered as the overall uptake) (see Methods). In the patients in whom this ratio could be evaluated (n = 30), it was significantly and inversely correlated with leucocytosis (r^2^ = 0.58), percentage of CD34^+^ cells in peripheral blood (r^2^ = 0.50), number of circulating CD34^+^ cells (r^2^ = 0.62), and Sokal prognostic score (r^2^ = 0.45).

## Discussion

In oncology, the ability of a targeted therapy to reach the target cell is an essential pharmacological step for effective targeting of malignant cells. CML is a model in this field with the development of anti-BCR-ABL TKIs and the possibility of identifying clone subpopulations by flow cytometry. We have adapted the original procedure described for imatinib^25^ to the detection of nilotinib, a second-generation TKI. After validating the procedure with three CML cell lines, we demonstrated that nilotinib can be detected in primary chronic phase-CML cells. In this controlled system, we observed significant differences in nilotinib uptake by PMN, monocytes and lymphocytes, with the highest values in PNM (i.e. the main cells in the CML clone). In these experiments, nilotinib uptake was essentially dependent on the intrinsic properties of the cells. The two-phase curve of in vitro nilotinib accumulation in CML cell lines and in primary cells suggests a first phase of fast, possibly active entry into the cell followed by a slower flow until the plateau that represents the system saturation. We found differences between nilotinib and imatinib^25^. Specifically, nilotinib accumulated less in KCL22 cells, and its uptake by monocytes was significantly higher than in lymphocytes in which both TKIs accumulate little, confirming different mechanisms of trans-membrane passage of the two TKIs^27^. These results might be partly related to the heterogeneous expression of membrane transporters by different cell types and the specific affinity of each TKI for these transporters. They could be consistent with the apparently contradictory results on the role of the influx transporter OCT1 in nilotinib intracellular penetration that was partially explained by its involvement in the early phase of penetration^28^, followed by its rapid saturation and inhibition^22^. However, the system is quite complex because nilotinib could be both a substrate and an inhibitor of the transporters ABCG2 and ABCB1^29–33^, and TKIs can modify also their membrane expression. Therefore the role of ATP-binding cassette transporters in the resistance to TKI, particularly nilotinib, remains debated and difficult to study^22,27,28,33,34^. For this reason, we decided to quantify TKI intracellular accumulation as the final outcome of these complex mechanisms.

Due to CML cell intra-clonal heterogeneity, and LSC resistance to TKIs, we compared the intracellular uptake of nilotinib in both CD34^+^ cells and PMN from the same patient. Nilotinib intracellular concentration in the immature CD34^+^ cell population was very variable, and it was undetectable in 40% of samples compared with 13.3% in PMN. This suggests that this immature subpopulation may be more resistant to nilotinib through its active rejection via a yet unknown mechanism due to the reported lack of involvement of the usual membrane transporters, such as ABCG2 that is strongly expressed by this subpopulation^22^. This result is consistent with the observed resistance to TKIs, including nilotinib, of CD34^+^ progenitor cells and CD34^+^ CD38^-^ LSC^35,36^. Unlike the interpatient heterogeneity, nilotinib accumulated homogeneously within each individual CD34^+^ cell sample without identification of a specific subpopulation (data not shown). This observation suggests a similar uptake by cycling and quiescent cells, or a very low/undetectable number of quiescent cells. However, as a previous study indicated that 10–20% of CD34^+^ cells are quiescent^35^, our flow cytometry approach should have detected them and therefore, it is likely that nilotinib penetrates into quiescent cells as well.

Furthermore, our sensitive single-cell technique showed that in some patients, nilotinib accumulates in CD34^+^ cells, sometimes at high level, and this should contribute to its targeting efficiency. This suggests individual patient’s mechanisms of regulation of nilotinib uptake that could partly explain why in some patients, TKI treatment can eliminate also immature CML cells, leading to long-term remission after treatment withdrawal. However, the analysis of the impact of intracellular nilotinib on BCR-ABL activity showed that for similar nilotinib intra-cellular concentrations, inhibition of TK activity was partial in CD34^+^ cells and almost complete in PMN. This is in agreement with previous findings^36^, but we could also demonstrate that a residual activity of the adaptor protein CrkL persisted despite significant nilotinib accumulation in the target cells. This could be explained by i) a limited efficacy of nilotinib, indicating the interest of combinatorial therapies with TKI molecules that target another pathway, such as asciminib, which binds to the myristoyl pocket of ABL in BCR-ABL instead of the ATP-binding domain^37^; ii) the existence in these more immature cells of BCR-ABL-independent CrkL activation pathways. For example, it has been shown that CrkL is engaged in type I interferon receptor signalling^38,39^, and is implicated in the signalling pathways of lymphoid cells (B- and T-cell receptors, IL-7…)^40,41^. However, the role of similar molecular mechanisms in CML cells is unknown. More, interestingly, CrkL is a TGF-β target in other cancer models^42^, but TGF-β is implicated in CML tumorigenicity and TKI resistance^43,44^.

Finally, in this small series, we analysed the relationship between intra-cellular nilotinib concentration and tumour burden in patients with chronic phase CML who received nilotinib (n = 33) or other TKI (n = 59) as first-line treatment. We found that nilotinib concentration in CD34^+^ cells was not correlated with the patient characteristics. Conversely, the relationship with the therapeutic response at month 3, 6 and 12 was almost significant, possibly due to lack of statistical power explained by the high number of patients in whom CD34^+^ cells did not accumulate nilotinib. On the other hand, nilotinib concentration in PMN was inversely correlated with leucocytosis, the percentage of CD34^+^ cells, the number of CD34^+^ cells per microlitre of blood, and the Sokal score. As at diagnosis, almost all CD34^+^ cells are Ph1-positive^45^ and their number correlates with the tumour mass and therapeutic response^46^, our findings indicate that nilotinib uptake by mature malignant cells is inversely correlated with the tumour mass. This observation was confirmed by the correlation of the intracellular nilotinib ratio with the Sokal score, leucocytosis and proportion of CD34^+^ cells in the clone. Specifically, in samples from patients with high Sokal score and tumour mass, nilotinib intracellular concentration was lower in PMN than in CD34^+^ cells. Overall, these results suggest that poor prognostic parameters at chronic phase-CML diagnosis are correlated with lower nilotinib uptake by mature cells in the clone that represent the vast majority of malignant cells at diagnosis. Therefore, significant uptake by this population could be an important parameter of nilotinib targeting efficiency related to the early therapeutic response. Indeed, the initial CML tumour mass is a parameter of disease aggressiveness and poor prognosis, which is partially described by the Sokal score. Overall, the initial tumour mass could be an indicator of the clone proliferative capacity and of the cellular properties that interfere with nilotinib intracellular uptake and consequently cell targeting efficacy, which is confirmed by the decrease in the circulating malignant clone after day 6 ± 1 of treatment. Thus, nilotinib penetration in PMN and not in CD34^+^ CML cells could play an important role in the early phases of the therapeutic response.

Given the already reported persistence of a small CD34^+^ cell population during treatment, the lack of correlation between nilotinib accumulation in CD34^+^ cells and the patient’s clinical features is surprising. The sample size in our study might have been too small to investigate this relationship due the high heterogeneity of nilotinib concentration in this population. However, therapeutic targeting of immature cells might be more complex and intracellular nilotinib concentration is only one of the involved parameters, which is generally accepted but has never been documented. The inter-patient variability of the intrinsic properties of CML CD34^+^ cells remains poorly explained.

Our study has some limitations. It was an in vitro study that evaluated only the intrinsic properties of cells in standardized conditions. It is likely that nilotinib uptake in vivo is not perfectly identical; however, the identification of a correlation between nilotinib concentration in PMN and patient prognostic criteria is in favour of a role of the intrinsic nilotinib uptake capacities of cells in its therapeutic efficacy. An in vivo study would be desirable, but technically difficult because of the low number of malignant cells that persist during treatment and the difficulty of distinguishing malignant cells from normal cells despite advances in immunophenotyping of immature cells using, for example, CD26 and CD93^47,48^.

In conclusion, the original procedure used to evaluate the intracellular accumulation of nilotinib allowed us to detect nilotinib in mature PMN and CD34^+^ cell subsets of the primary CP-CML clone in standardized conditions. For the first time, we could show the inter-individual variability and the intra-clonal heterogeneity of nilotinib accumulation, but no relationship between nilotinib intracellular concentration in the two subpopulations. We also revealed the absence of nilotinib uptake by CD34^+^ target cells in some patients and much more frequently (3 times more) than in PMN. Furthermore, we confirmed that BCR-ABL inhibition is partial in chronic phase-CML CD34^+^ cells that accumulated a significant amount of nilotinib, suggesting a lower targeting efficacy of nilotinib in this subset through yet unknown mechanisms. However, nilotinib uptake by CD34^+^ cells was not related to the CML characteristics, Sokal prognostic score, or clone decrease after 6 ± 1 days of therapy. Conversely, nilotinib uptake by PMN was inversely correlated with these parameters, suggesting a negative relationship between the intrinsic capacity of PMN to accumulate nilotinib and the CML clone proliferative capacity. Nilotinib accumulation in mature cells rather than in CD34^+^ cells might influence the early therapeutic response. Given the long-term persistence of immature cells, assessing the possible relationship between nilotinib concentration in CD34^+^ cells and long-term therapeutic response would require specific studies in this subpopulation. The ability of chronic phase CML cells to accumulate nilotinib is probably a crucial step in its targeting efficiency and additional studies are needed to understand the underlying mechanisms.

## Methods

### Cell lines and primary cells

The BCR-ABL-positive K562, KCL22 and LAMA84 cell lines were derived from patients with CML in blast crisis. The K562 and LAMA84 cell lines were purchased from ATCC (Molsheim, France), and the KCL22 line was kindly provided by Dr V. Maguer Satta (UMR INSERM 1052 CNRS 5286 Centre Léon Bérard, Lyon). K562 and LAMA84 cells were grown in Iscove’s modified Dulbecco’s medium, and KCL22 cells in RPMI 1640 (Lonza, Verviers, Belgium). All cell lines were cultured in a humidified incubator at 37 °C in an atmosphere of 5% CO_2_. All experiments were done with cells in the log phase of growth.

Blood samples from patients with CML in chronic phase were collected in lithium heparin tubes at diagnosis, before any treatment (n = 92 patients). Nucleated cells were isolated by collecting the buffy coat, and erythrocytes were lysed using ammonium chloride (Stemcell Technologies, Vancouver, Canada). Cells were counted and plated at 1 × 10^6^ cells per millilitre in minimal essential medium (Lonza) supplemented with 4% foetal calf serum. All experiments were carried out with fresh cells, within 24 h of sampling.

Clinical and laboratory data were collected at diagnosis for all patients, and hemogram parameters at day 6 ± 1 after NIL initiation. Residual disease levels were available at 3 (n = 72 for all patients, n = 30 for patients with NIL), 6 (n = 75 and n = 31 for all patients and patients with NIL, respectively), and 12 months (n = 70 and n = 27 for all patients and patients with NIL, respectively). Written informed consent was obtained from all patients, and the study was approved by the local ethics committee (C.P.P. Ouest V, CHU Pontchaillou, 9 Avenue Bataille Flandre-Dunkerque 35,033 Rennes Cedex 9).

### Nilotinib solubilization

Nilotinib (Sequoia Research Product, Pangbourne, UK) was dissolved in sterile DMSO. Stock solutions were prepared at 10 mM, aliquoted, and kept at -20 °C until use.

### Flow cytometry analysis of intracellular nilotinib level

Nilotinib intracellular level was measured by flow cytometry using a BD FACSAria SORP -flow cytometer (Becton Dickinson, Le Pont de Claix, France) equipped with a Genesis G2 355 nm laser (Coherent, Orsay, France), used at a power supply of 100 mWatt. UV fluorescence was detected using a 450/50 Band Pass filter.

As nilotinib is naturally fluorescent under UV light, for its intracellular level quantification it was assumed that in a controlled system, the UV fluorescence difference between control (untreated) and cells incubated with nilotinib was directly correlated to the additional amount of fluorescent nilotinib taken up by the cell. As many cellular components have intrinsic fluorescence and each cell has a weak natural fluorescence under UV light, it was essential to pre-determine the amount of fluorescent light emitted by each cell population. Therefore, nilotinib intracellular concentration was defined as the additional fluorescence units (AFU) relative to control cells.

BD FACSDiva CS&T Research calibrated beads were used to monitor the cytometer performance each day in order to generate reproducible data.

### Assessment of the in vitro kinetics of nilotinib uptake by flow cytometry

Nilotinib uptake kinetics was assessed by measuring the cell UV fluorescence at 5, 15, 30, 60, 120 and 240 min of incubation with nilotinib at two different doses (1 and 5 µM) in the usual cell culture medium. At each time point, nilotinib uptake was stopped by putting the tubes on ice. Then, cells were analysed by flow cytometry after addition of propidium iodide (PI) (2 µL per 5 × 10^5^ cells) to identify viable (PI negative) cells. To limit the variability linked to cell morphology/linages of patient CML samples, three immortalized CML cell lines (K562, KCL22 and LAMA84) were used.

### Correlation between nilotinib intracellular levels measured by flow cytometry and by physical–chemical assay

To evaluate the correlation between flow cytometry and high-performance liquid chromatography (HPLC; a standard analytical method) data, nilotinib-related UV fluorescence in each cell and the nilotinib amount released after lysis of a known number of cells from the same cell suspension sample were quantified. To this aim, a defined number (5 × 10^6^) of K562 or LAMA84 cells were incubated with different concentrations of nilotinib (0.02, 0.5, 1 and 2.5 µM) at 37 °C in a saturated humidified atmosphere of 5% CO_2_ for 2 h. After stopping nilotinib uptake by diluting the cell suspension with cold medium, samples were washed twice and kept on ice. After the last wash, 150 µL of cell suspension was used for measuring nilotinib intracellular level flow cytometry. The other cell fraction was used for the physical–chemical assay. Specifically, cells were counted and viability was evaluated by trypan blue exclusion. After removal of as much supernatant as possible, cell pellets were stored at -80 °C until analysis (Dr MC Gagnieu’s Laboratory, Lyon, France). After cell lysis (liquid-based homogenization), nilotinib was quantified (pg/cell) by HPLC with a UV diode array detector, using three wavelengths (264, 240 and 290 nm). A spectral analysis was performed to ensure the purity of the chromatographic peaks.

### Correlation between extra- and intra-cellular amount of nilotinib after 2 h of incubation

Cells (CML cell lines and primary cells) were incubated with increasing concentrations of nilotinib for 2 h. In primary cell samples, lymphocytes, monocytes, and polymorphonuclear cells were identified on the basis of their forward and low side light scattering characteristics. At least 50 000 target events were acquired and analysed. To analyse CD34^+^ cells, samples were first incubated with 5 µl of anti-CD34-FITC and 5 µl of anti-CD38-PE antibodies (Becton Dickinson, Le Pont de Claix, France) at room temperature for 20 min, and then washed once in 1 mL of PBS/1% bovine serum albumin.

### ICNIL ratio calculation

To take into account the differences in nilotinib uptake between CD34^+^ cells and mature cells (PNM) from the same clone, each cell type was considered to be representative of a cell compartment. Therefore, the intra-cellular NIL (ICNIL) ratio was calculated as the difference in mean intracellular nilotinib concentration between CD34^+^ cells and PMN divided by the sum of the mean intracellular nilotinib concentration in CD34^+^ cells and PMN: ICNIL = [ICNIL]34—[ICNIL]^PMN^ / [ICNIL]34 + [ICNIL]^PMN^. Consequently, the ratio values are distributed between -1 (cell uptake only by PMN) and 1 (cell uptake only by CD34^+^ cells). The mean intracellular nilotinib concentration in each cell type was considered to be representative of the capacity of the single cells in the sample to accumulate nilotinib.

### Assessment of CrkL phosphorylation by flow cytometry

CrkL phosphorylation was evaluated in K562 cells and in primary mature and immature CD34^+^ CML cells from patients in chronic phase at diagnosis (before any treatment) using the method described by Hamilton A. et al^49^. Briefly, 1 × 10^6^ cells were fixed using BD Fix and Lyse buffer, and then permeabilized with BD Phosflow Perm Buffer III (Becton Dickinson, Le Pont de Claix, France). Cells were washed twice with Stain Buffer and incubated with an anti-phosphorylated CrkL antibody or isotype matched control (Becton Dickinson) at room temperature for 1 h. Cells were then washed with Stain Buffer and PBS before analysis. Results were expressed as the ratio between the mean fluorescence intensity (MFI) of the labelled sample and of the isotype control.

### Statistical analyses

Statistical analyses were performed using the Stata software, version 15 (StataCorp, College Station, US). Tests were two-sided, with a type I error set at 5%. Continuous data were expressed as the mean and standard deviation or median and [interquartile range] according to their statistical distribution. The assumption of normality was assessed with the Shapiro–Wilk test. Continuous variables were compared between groups with the Student’s *t*-test or the Mann–Whitney test, when the assumptions of the *t*-test were not met. Homoscedasticity was analysed using the Fisher-Snedecor’s test. Categorical parameters were compared between groups using the chi-square or Fisher’s exact test. Relationships between continuous variables were assessed by estimating the Pearson or Spearman correlation coefficients, according to their statistical distribution, with the Sidak’s type I error correction due to multiple comparisons. Random effects models were used to measure the correlation between intracellular and extracellular nilotinib concentrations, taking into account the between and within patient variability. The normality of residuals from these models was assessed as aforementioned. When appropriate, data were logarithmically transformed to achieve the normality of dependent outcomes.

## Supplementary Information

Supplementary Information

## Abbreviations

CML: Chronic myeloid leukaemia
ICNIL: Intracellular Nilotinib
TKI: Tyrosine Kinase Inhibitors
CP-CML: Chronic phase-chronic myeloid leukaemia
LSC: Leukemic stem cells

## References

[1] Morgan P (2012). Can the flow of medicines be improved? Fundamental pharmacokinetic and pharmacological principles toward improving Phase II survival. Drug Discov. Today.

[2] Hann MM, Simpson GL (2014). Intracellular drug concentration and disposition–the missing link?. Methods San Diego Calif..

[3] Lin P-J (2016). Linking costs and survival in the treatment of older adults with chronic myeloid leukemia: an analysis of SEER-medicare data from 1995 to 2007. Med. Care.

[4] Steegmann JL (2016). European LeukemiaNet recommendations for the management and avoidance of adverse events of treatment in chronic myeloid leukaemia. Leukemia.

[5] Kalmanti L (2015). Safety and efficacy of imatinib in CML over a period of 10 years: data from the randomized CML-study IV. Leukemia.

[6] Druker BJ (1996). Effects of a selective inhibitor of the Abl tyrosine kinase on the growth of Bcr-Abl positive cells. Nat. Med..

[7] Hochhaus A (2017). Long-term outcomes of imatinib treatment for chronic Myeloid Leukemia. N. Engl. J. Med..

[8] Hochhaus A (2020). Expert opinion-management of chronic myeloid leukemia after resistance to second-generation tyrosine kinase inhibitors. Leukemia.

[9] Mahon F-X (2010). Discontinuation of imatinib in patients with chronic myeloid leukaemia who have maintained complete molecular remission for at least 2 years: the prospective, multicentre Stop Imatinib (STIM) trial. Lancet Oncol..

[10] Chomel J-C (2011). Leukemic stem cell persistence in chronic myeloid leukemia patients with sustained undetectable molecular residual disease. Blood.

[11] Chomel JC (2016). Leukemic stem cell persistence in chronic myeloid leukemia patients in deep molecular response induced by tyrosine kinase inhibitors and the impact of therapy discontinuation. Oncotarget.

[12] Weisberg E (2005). Characterization of AMN107, a selective inhibitor of native and mutant Bcr-Abl. Cancer Cell.

[13] Cortes JE (2016). Final 5-year study results of DASISION: the dasatinib versus imatinib study in treatment-naïve chronic Myeloid Leukemia patients trial. J. Clin. Oncol. Off. J. Am. Soc. Clin. Oncol..

[14] Hochhaus A (2016). Long-term benefits and risks of frontline nilotinib vs imatinib for chronic myeloid leukemia in chronic phase: 5-year update of the randomized ENESTnd trial. Leukemia.

[15] Rosti G (2009). Nilotinib for the frontline treatment of Ph(+) chronic myeloid leukemia. Blood.

[16] Saglio G (2010). Nilotinib versus imatinib for newly diagnosed chronic myeloid leukemia. N. Engl. J. Med..

[17] Kantarjian HM (2011). Nilotinib versus imatinib for the treatment of patients with newly diagnosed chronic phase, Philadelphia chromosome-positive, chronic myeloid leukaemia: 24-month minimum follow-up of the phase 3 randomised ENESTnd trial. Lancet Oncol..

[18] Kantarjian HM (2011). Nilotinib is effective in patients with chronic myeloid leukemia in chronic phase after imatinib resistance or intolerance: 24-month follow-up results. Blood.

[19] Tanaka C (2010). Clinical pharmacokinetics of the BCR-ABL tyrosine kinase inhibitor nilotinib. Clin. Pharmacol. Ther..

[20] Giles FJ (2013). Nilotinib population pharmacokinetics and exposure-response analysis in patients with imatinib-resistant or-intolerant chronic myeloid leukemia. Eur. J. Clin. Pharmacol..

[21] Larson RA (2012). Population pharmacokinetic and exposure-response analysis of nilotinib in patients with newly diagnosed Ph+ chronic myeloid leukemia in chronic phase. Eur. J. Clin. Pharmacol..

[22] Davies A (2009). Nilotinib concentration in cell lines and primary CD34(+) chronic myeloid leukemia cells is not mediated by active uptake or efflux by major drug transporters. Leukemia.

[23] Shukla S (2011). Synthesis and characterization of a BODIPY conjugate of the BCR-ABL kinase inhibitor Tasigna (nilotinib): evidence for transport of Tasigna and its fluorescent derivative by ABC drug transporters. Mol. Pharm..

[24] Maia RC, Vasconcelos FC, Souza PS, Rumjanek VM (2018). Towards comprehension of the ABCB1/P-glycoprotein role in chronic Myeloid Leukemia. Mol. Basel Switz..

[25] Bourgne C (2012). Measurement of imatinib uptake by flow cytometry. Cytom Part J. Int. Soc. Anal. Cytol..

[26] Monici M (2005). Cell and tissue autofluorescence research and diagnostic applications. Biotechnol. Annu. Rev..

[27] Polillo M (2015). Pharmacogenetics of BCR/ABL inhibitors in chronic Myeloid Leukemia. Int. J. Mol. Sci..

[28] Yamakawa Y (2014). Distinct interaction of nilotinib and imatinib with P-Glycoprotein in intracellular accumulation and cytotoxicity in CML Cell Line K562 cells. Biol. Pharm. Bull..

[29] Kosztyu P, Bukvova R, Dolezel P, Mlejnek P (2014). Resistance to daunorubicin, imatinib, or nilotinib depends on expression levels of ABCB1 and ABCG2 in human leukemia cells. Chem. Biol. Interact..

[30] Hegedus C (2009). Interaction of nilotinib, dasatinib and bosutinib with ABCB1 and ABCG2: implications for altered anti-cancer effects and pharmacological properties. Br. J. Pharmacol..

[31] White DL (2006). OCT-1-mediated influx is a key determinant of the intracellular uptake of imatinib but not nilotinib (AMN107): reduced OCT-1 activity is the cause of low in vitro sensitivity to imatinib. Blood.

[32] Brendel C (2007). Imatinib mesylate and nilotinib (AMN107) exhibit high-affinity interaction with ABCG2 on primitive hematopoietic stem cells. Leukemia.

[33] Dohse M (2010). Comparison of ATP-binding cassette transporter interactions with the tyrosine kinase inhibitors imatinib, nilotinib, and dasatinib. Drug Metab. Dispos. Biol. Fate Chem..

[34] Mahon F-X (2008). Evidence that resistance to nilotinib may be due to BCR-ABL, Pgp, or Src kinase overexpression. Cancer Res..

[35] Graham SM (2002). Primitive, quiescent, Philadelphia-positive stem cells from patients with chronic myeloid leukemia are insensitive to STI571 in vitro. Blood.

[36] Jørgensen HG, Allan EK, Jordanides NE, Mountford JC, Holyoake TL (2007). Nilotinib exerts equipotent antiproliferative effects to imatinib and does not induce apoptosis in CD34^+^ CML cells. Blood.

[37] Schoepfer J (2018). Discovery of Asciminib (ABL001), an allosteric inhibitor of the tyrosine kinase activity of BCR-ABL1. J. Med. Chem..

[38] Ahmad S, Alsayed YM, Druker BJ, Platanias LC (1997). The type I interferon receptor mediates tyrosine phosphorylation of the CrkL adaptor protein. J. Biol. Chem..

[39] Platanias LC (2005). Mechanisms of type-I- and type-II-interferon-mediated signalling. Nat. Rev. Immunol..

[40] Lauenstein JU (2019). Phosphorylation of the multifunctional signal transducer B-cell adaptor protein (BCAP) promotes recruitment of multiple SH2/SH3 proteins including GRB2. J. Biol. Chem..

[41] Aiello FB (2018). IL-7-induced phosphorylation of the adaptor Crk-like and other targets. Cell. Signal..

[42] Cheng S, Guo J, Yang Q, Han L (2015). Crk-like adapter protein is required for TGF-β-induced AKT and ERK-signaling pathway in epithelial ovarian carcinomas. Tumour Biol. J. Int. Soc. Oncodevelopmental Biol. Med..

[43] Naka K (2010). TGF-beta-FOXO signalling maintains leukaemia-initiating cells in chronic myeloid leukaemia. Nature.

[44] Naka K (2016). Novel oral transforming growth factor-β signaling inhibitor EW-7197 eradicates CML-initiating cells. Cancer Sci..

[45] Thielen N (2016). Leukemic stem cell quantification in newly diagnosed patients with chronic Myeloid Leukemia predicts response to nilotinib therapy. Clin. Cancer Res Off. J. Am. Assoc. Cancer Res..

[46] Mustjoki S (2013). Impact of malignant stem cell burden on therapy outcome in newly diagnosed chronic myeloid leukemia patients. Leukemia.

[47] Bocchia M (2018). Residual peripheral blood CD26+ Leukemic stem cells in chronic myeloid leukemia patients during TKI therapy and during treatment-free remission. Front. Oncol..

[48] Kinstrie R (2020). CD93 is expressed on chronic myeloid leukemia stem cells and identifies a quiescent population which persists after tyrosine kinase inhibitor therapy. Leukemia.

[49] Hamilton A (2006). BCR-ABL activity and its response to drugs can be determined in CD34^+^ CML stem cells by CrkL phosphorylation status using flow cytometry. Leukemia.

