# Supplementary material for: Efficiency of nilotinib to target chronic phase-chronic myeloid leukaemia primary mature CD34− and immature CD34+ cells
Source: Sci Rep. 2021 Mar 17;11:6187. doi: 10.1038/s41598-021-85734-0 (PMC7969931; doi:10.1038/s41598-021-85734-0)

**Efficiency of nilotinib to target chronic phase-chronic myeloid leukaemia primary mature CD34- and immature CD34+ cells**

Marc G Berger^1,2,3,11^ , Benjamin Lebecque^1,2^, Thomas Tassin^1,2^, Louis-Thomas Dannus^1,2^, Juliette Berger^1,2,3^, Mélanie Soucal^1,2^, Agnès Guerci^4^, Pascale Cony-Makhoul^5^, Hyacinthe Johnson^6^, Gabriel Etienne^7^, Denis Guyotat^8^, Marie-Claude Gagnieu^9^, Bruno Pereira^10^, Sandrine Saugues^2,3^, Olivier Tournilhac^2,11^, Eric Hermet^11^, and Céline Bourgne^1,2^

^1^Hématologie Biologique, CHU Clermont-Ferrand, Hôpital Estaing, 1 place Lucie et Raymond Aubrac, 63003 Clermont-Ferrand Cedex 1, France

^2^Equipe d’Accueil 7453 CHELTER, Université Clermont Auvergne, CHU Clermont-Ferrand, Hôpital Estaing, 1 place Lucie et Raymond Aubrac, 63003 Clermont-Ferrand Cedex 1, France

^3^CRB-Auvergne, CHU Clermont-Ferrand, Hôpital Estaing, 1 place Lucie et Raymond Aubrac, 63003 Clermont-Ferrand Cedex 1, France

^4^Hématologie Clinique, CHU Nancy, Hôpitaux de Brabois, Rue du Morvan, 54500 Vandoeuvre-lès-Nancy, France

^5^Hématologie Clinique, CH Annecy-Genevois, 1 Avenue de l’Hôpital, 74370 Metz-Tessy, France

^6^Institut d’Hématologie de Basse Normandie, CHU de Caen, Avenue de la Côte de Nacre, CS30001, 14033 Caen Cedex 9, France

^7^Hématologie Clinique, Institut Bergonié, 229 Cours de l’Argonne, 33076 Bordeaux Cedex, France

^8^Département d'Hématologie, Institut de Cancérologie Lucien Neuwirth, 108 Avenue Albert Raimond, 42270 Saint-Priest-en-Jarez, France

^9^Service de Biochimie et Biologie Moléculaire, UM Pharmacologie-Toxicologie, Groupement Hospitalier Sud,165, chemin du grand Revoyet, 69495 Pierre-Bénite, France

^10^CHU Clermont-Ferrand, Délégation à la Recherche Clinique et à l'Innovation, 63003, Clermont-Ferrand, France

^11^Hématologie Clinique, CHU Clermont-Ferrand, Hôpital Estaing, 1 place Lucie et Raymond Aubrac, 63003 Clermont-Ferrand, France

**Running Title:** Flow cytometry evaluation of nilotinib uptake by CML cells

**Correspondence:** Dr Céline BOURGNE (PhD), Equipe d’Accueil 7453 CHELTER, Université Clermont Auvergne, Service Hématologie Biologique, CHU Estaing, 1 place Lucie et Raymond Aubrac, 63003 CLERMONT-FERRAND Cedex 1, France.

Tel: (33) 4 73 75 02 00. Fax: (33) 4 73 75 02 15. E-mail: [cbourgne@chu-clermontferrand.fr](mailto:cbourgne@chu-clermontferrand.fr)


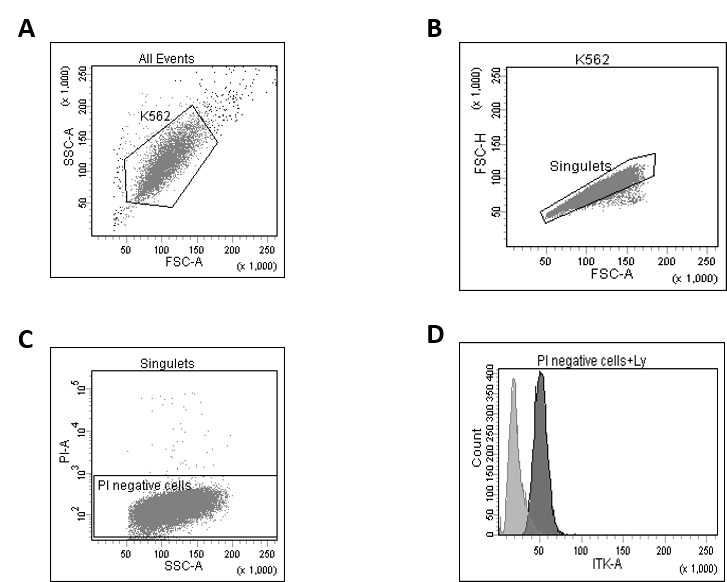


**Supplementary Figure S1: Measurement of nilotinib uptake by K562 cells using flow cytometry**

A flow cytometer equipped with a UV-laser was used to detect the fluorescence emitted by nilotinib. K562 cells were first gated using a FSC-SSC dot-plot (**A**). Then, singlets were selected (FSC-H *vs* FSC-A) (**B**) before selection of propidium iodide-negative (live) cells (**C**). The difference in the fluorescence mean intensity (linear scale) between control (untreated) and cells incubated with nilotinib was used to quantify the amount of intracellular nilotinib (**D**).

**
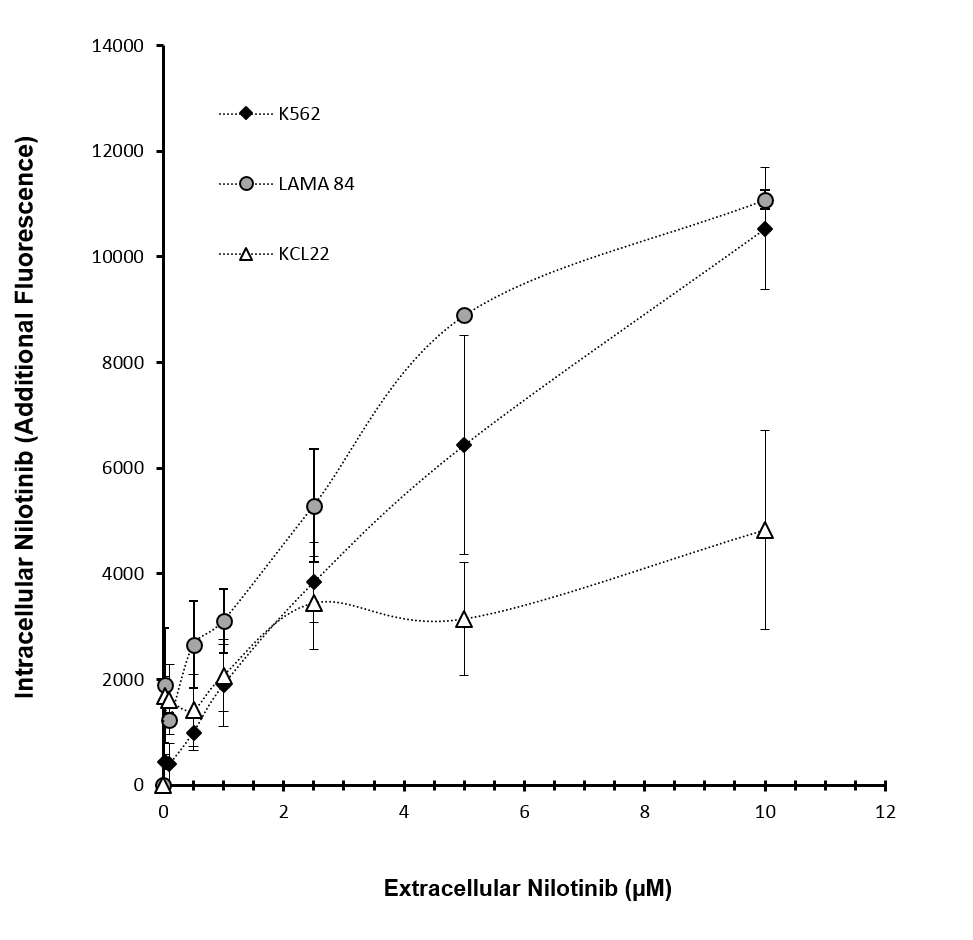
**

**Supplementary Figure S2: Relationship between intra- and extra-cellular amount of nilotinib in CML cell lines.**

Nilotinib accumulation was significantly different in the three CML-derived cell lines tested (K562, KCL22 and LAMA84). When incubated with low nilotinib concentrations (0.5 µM; extracellular nilotinib), nilotinib intracellular level was significantly higher in LAMA84 than in K562 cells (2.7x10^3^ ± 0.8 x10^3^ *vs* 1x10^3^ ± 0.3 x10^3^ AFU; p=0.05). Nilotinib intracellular concentration in KCL22 cells reached a plateau when cells were incubated with 2.5 μM of nilotinib, and in K562 and LAMA84 after incubation with 5 μM. Results are the mean ± standard deviation.

**Supplementary Table 1: Patients characteristics**


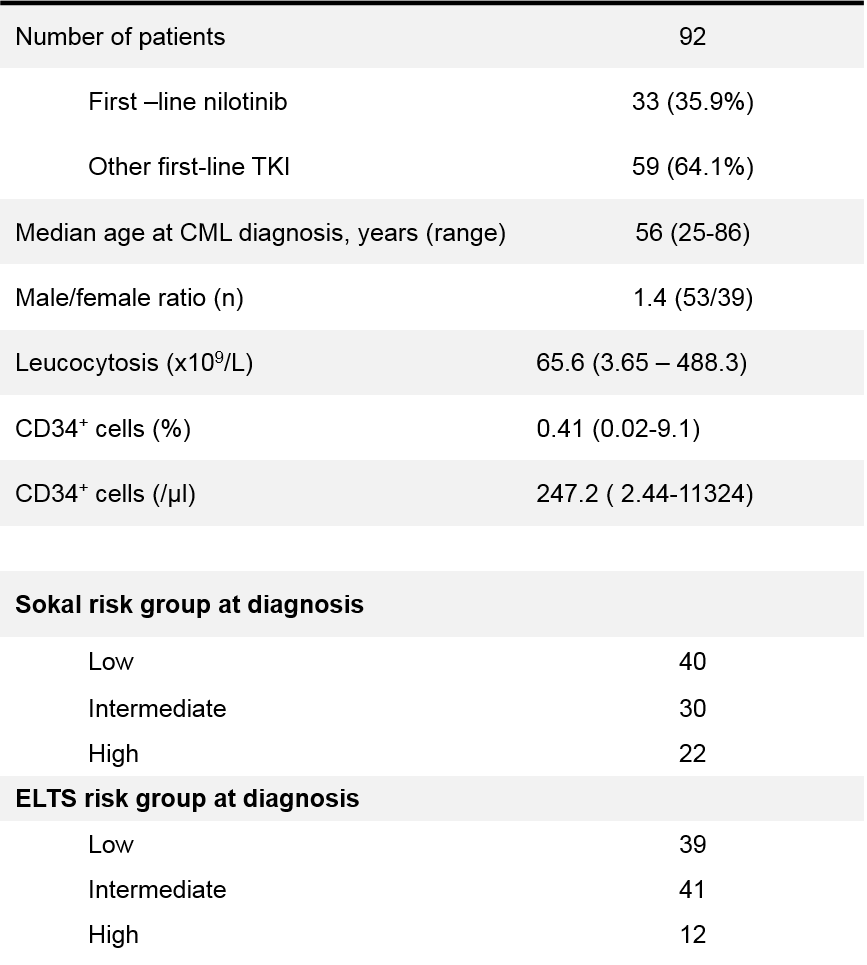


**Supplementary Table 2 : Relationship between intra- and extra-cellular amount of nilotinib in primary CML cells.**

|  | 0 | 0.1 µM | 1 µM | 2.5 µM | 5 µM |
| --- | --- | --- | --- | --- | --- |
| Ly | 0 | 0.029±0.005 | 0.031±0.005 | 0.045±0.006 | 0.011±0.01 |
| Mo | 0 | 0.099±0.02 | 0.13±0.02 | 0.17±0.02 | 0.33±0.03 |
| PMN | 0 | 0.13±0.02 | 0.20±0.02 | 0.31±0.03 | 0.54±0.05 |

Nilotinib uptake by primary cells was evaluated by flow cytometry after 2 hours of incubation with 0.1, 1, 2.5 or 5µM of this TKI (n=60 patients). Lymphocytes (Ly), monocytes (Mo), and polymorphonuclear cells (PMN) were identified on the basis of their FSC/SSC parameters. Data are expressed as the mean ± standard deviation

**Supplementary Table 3: Characteristics of patients who received nilotinib as first-line treatment**


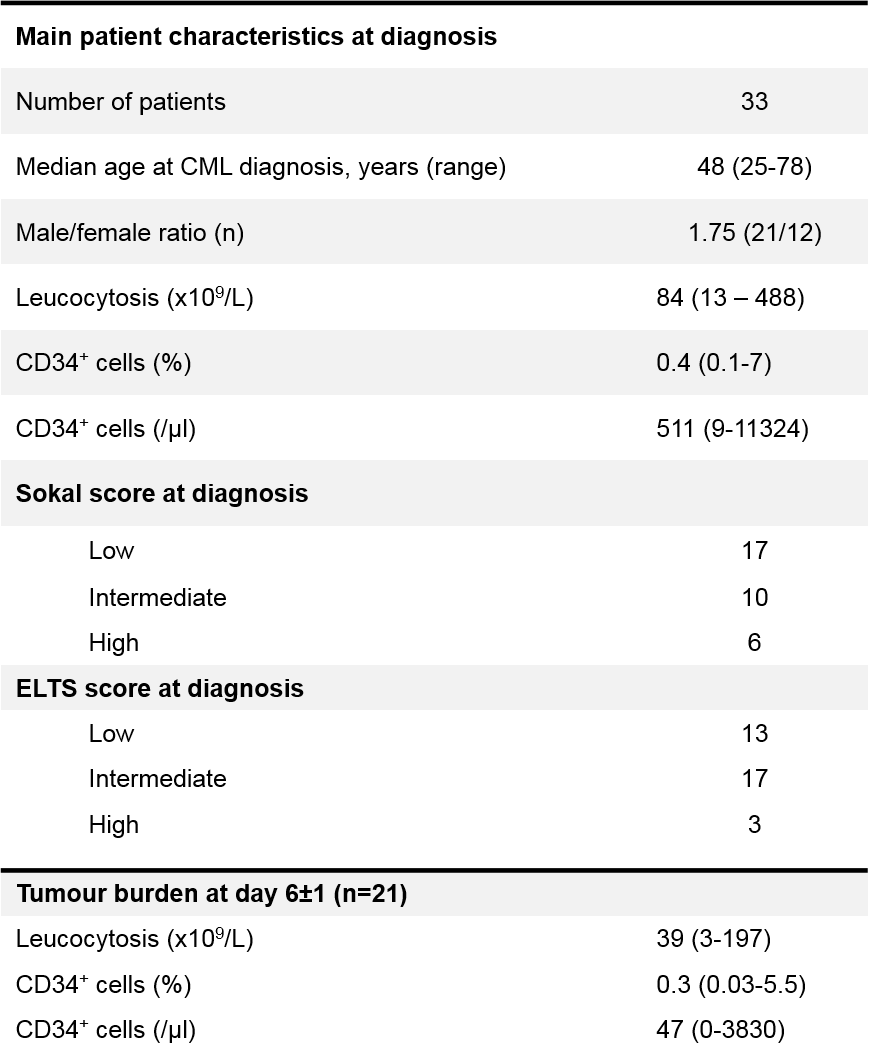

Supplement: Supplementary file 1 — Supplementary Information [file 41598_2021_85734_MOESM1_ESM.docx]
